# Sporadic clone *Escherichia coli* ST615 as a vector and reservoir for dissemination of crucial antimicrobial resistance genes

**DOI:** 10.3389/fcimb.2024.1368622

**Published:** 2024-04-29

**Authors:** Laura Camila Carrera Páez, Martin Olivier, Anahí Samanta Gambino, Tomás Poklepovich, Andrea Pamela Aguilar, María Paula Quiroga, Daniela Centrón

**Affiliations:** ^1^ Laboratorio de Investigaciones en Mecanismos de Resistencia a Antibióticos, Instituto de Investigaciones en Microbiología y Parasitología Médica, Facultad de Medicina, Universidad de Buenos Aires - Consejo Nacional de Investigaciones Científicas y Tecnológicas (IMPaM, UBA-CONICET), Buenos Aires, Argentina; ^2^ The Research Institute of the McGill University Health Centre, McGill University, Montréal, QC, Canada; ^3^ Plataforma de Genómica y Bioinformática, Instituto Nacional de Enfermedades Infecciosas - La Administración Nacional de Laboratorios e Institutos de Salud (INEI-ANLIS) “Dr. Carlos G. Malbrán”, Buenos Aires, Argentina; ^4^ Clúster de Bioinformática, Instituto de Investigaciones en Microbiología y Parasitología Médica, Facultad de Medicina, Universidad de Buenos Aires - Consejo Nacional de Investigaciones Científicas y Tecnológicas (IMPaM, UBACONICET), Buenos Aires, Argentina

**Keywords:** antimicrobial resistance, conjugation, *mcr-1* gene, *Escherichia coli*, extracellular vesicles (EVs)

## Abstract

There is scarce information concerning the role of sporadic clones in the dissemination of antimicrobial resistance genes (ARGs) within the nosocomial niche. We confirmed that the clinical *Escherichia coli* M19736 ST615 strain, one of the first isolates of Latin America that harbors a plasmid with an *mcr-1* gene, could receive crucial ARG by transformation and conjugation using as donors critical plasmids that harbor *bla*_CTX-M-15_, *bla*_KPC-2_, *bla*_NDM-5_, *bla*_NDM-1_, or *aadB* genes. *Escherichia coli* M19736 acquired *bla*_CTX-M-15_, *bla*_KPC-2_, *bla*_NDM-5_, *bla*_NDM-1_, and *aadB* genes, being only blaNDM-1 maintained at 100% on the 10th day of subculture. In addition, when the evolved MDR-*E. coli* M19736 acquired sequentially *bla*_CTX-M-15_ and *bla*_NDM-1_ genes, the maintenance pattern of the plasmids changed. In addition, when the evolved XDR-*E. coli* M19736 acquired in an ulterior step the paadB plasmid, a different pattern of the plasmid’s maintenance was found. Interestingly, the evolved *E. coli* M19736 strains disseminated simultaneously the acquired conjugative plasmids in different combinations though selection was ceftazidime in all cases. Finally, we isolated and characterized the extracellular vesicles (EVs) from the native and evolved XDR-*E. coli* M19736 strains. Interestingly, EVs from the evolved XDR-*E. coli* M19736 harbored *bla*_CTX-M-15_ though the pDCAG1-CTX-M-15 was previously lost as shown by WGS and experiments, suggesting that EV could be a relevant reservoir of ARG for susceptible bacteria. These results evidenced the genetic plasticity of a sporadic clone of *E. coli* such as ST615 that could play a relevant transitional link in the clinical dynamics and evolution to multidrug/extensively/pandrug-resistant phenotypes of superbugs within the nosocomial niche by acting simultaneously as a vector and reservoir of multiple ARGs which later could be disseminated.

## 1 Introduction

*Escherichia coli* is common among the aerobic bacteria in the gastrointestinal tract microbiota of both humans and mammals (Denamur et al., 2021). Simultaneously, some lineages have developed into a pathogen well adapted to their host causing different diseases (Geurtsen et al., 2022), including adaptation to the nosocomial niche as high-risk clones or “superbugs” that rapidly evolve to extreme drug resistance (XDR). The lineage represented by sequence type (ST) 131 is the predominant isolate of hospital infections worldwide among *E. coli* strains that behave like an epidemic clone, also known as pandemic clones (Pitout and DeVinney, 2017; Soncini et al., 2022; Pitout and Chen, 2023). On the other hand, little is known about the role of sporadic clones of this species in the adaptation to multidrug resistance among clinical isolates. Since a few strains unrelated to outbreaks of *E. coli* ST615 have been reported from Tunisia (Maamar, 2016), Poland (Jamborova et al., 2015), and Spain (Ojer-Usoz et al., 2017) and *E. coli* M19736 from Argentina (Tijet et al., 2017), this ST may be considered as a sporadic clone. According to the World Health Organization, multidrug resistance (MDR) in Gram-negative bacilli (Gnb) has become a challenge due to its high global incidence and prevalence. In 2019, it was estimated that 4.95 million deaths were due to infections associated with antimicrobial resistance (AMR) (Global antimicrobial resistance and use surveillance system (GLASS) report: 2022, 2022). These pathogens represent a particular threat in nosocomial infections, and among those of greatest clinical interest, *Acinetobacter baumannii*, *Pseudomonas aeruginosa*, and *Enterobacteriaceae* producers of carbapenemases have been identified as a critical priority (Rello et al., 2019). MDR Gnb usually harbor multiple mobile genetic elements such as gene cassettes, transposons, and plasmids that confer their MDR phenotypes. These mobile genetic elements can be transferred by conjugation, transformation, and transduction and by the most recently discovered mechanism known as vesiduction through the extracellular vesicles (EVs).

The rapid increase of carbapenem-resistant Gnb due to the expression of enzymes such as KPC-2 (*Klebsiella pneumoniae* carbapenemase-2) and NDM-1 (New Delhi metallo-β-lactamase-1) is a global public health concern. Consequently, interest in another family of antibiotics, polymyxins, has recently increased as a last resort used in medical clinics despite its high nephrotoxicity (Rodríguez et al., 2018; Binsker et al., 2022). Furthermore, the intensive use of polymyxins in veterinary medicine not only for the treatment of infections but also as a growth promoter has led to an increase of the isolation of Gnb strains resistant to this antibiotic in clinical settings (Binsker et al., 2022). Resistance to polymyxins includes chromosome-encoded resistance traits, as well as the mobile plasmid-encoded polymyxin resistance determinants such as the *mcr-1* gene (Liu et al., 2016; Caniaux et al., 2017). The transferable *mcr-*1 gene was first detected in *E. coli* isolates from animals, food, and patients in China (Liu et al., 2016). Aside from this gene, 10 other *mcr-*like genes (from *mcr-2* to *mcr-10*) as well as several of their variants designated as *mcr-1.2*, *mcr-1.3*, *mcr-1.12*, etc. have been identified (Hussein et al., 2021). The *bla*_KPC_, *bla*_NDM_, and *mcr*-like genes are usually found in conjugative plasmids of diverse incompatibility groups, which enhance the challenge to combat the bacteria possessing these antibiotic-resistant determinants (Brandt et al., 2019; Cejas et al., 2022; Knecht et al., 2022; Li C. et al., 2022; Molina et al., 2022; Sanz et al., 2022; Boutzoukas et al., 2023; Riccobono et al., 2023). Despite the key role that conjugative plasmids have in nosocomial isolates, understanding how they can persist in bacterial populations in the absence of positive selection is challenging for pandemic and sporadic clones.

The nanosize EV entities secreted by Gnb have been recognized for their cardinal importance in intercellular communication among cells and to be responsible for the modulation of various biological functions (McMillan and Kuehn, 2021; Toyofuku et al., 2023). EVs are now well recognized for their role as long-distance secretion–delivery systems that eliminate the need for cell–cell contact (Toyofuku et al., 2023). EVs transport, harbor, and deliver in a concentrated, protected, and directed way biologically active proteins, lipids, nucleic acids, metabolites, and virulence factors between two bacterial cells (Tran and Boedicker, 2017; McMillan and Kuehn, 2021; Toyofuku et al., 2023). The exchange of DNA mediated by EV has been identified as an additional form of horizontal genetic transfer (HGT) (Chatterjee et al., 2017). It has been reported that they can carry DNA associated with the membrane and protect luminal genetic material against DNases and RNases (Kim et al., 2015). Of the DNA that is transferred, there are genes associated with AMR, which has huge clinical implications since the potential of propagation of a gene depends to a great extent on the competence to transmit (Chatterjee et al., 2017; Wagner et al., 2018). Earlier studies have reported the transfer by EV of a penicillin resistance gene in *Neisseria gonorrhoeae* (Dorward et al., 1989); the *bla*_OXA-24_ (Rumbo et al., 2011) and the *bla*_NDM-1_ carbapenemase genes in *Acinetobacter baumannii* (Chatterjee et al., 2017); the *bla*_CTX-M-15_ and *bla*_TEM-1_ genes in *E. coli* O104:H4 (Bielaszewska et al., 2020); and finally, the *bla*_NDM_, *bla*_KPC_, *bla*_SHV_, *bla*_CTX-M-9_, and *aac(6′)-Ib* genes in *K. pneumoniae* strains (Li P. et al., 2022). In the present study, we wonder about the ability of the *E. coli* M19736 clinical strain belonging to the sporadic clone ST615, which was one of the first isolates harboring the *mcr-1* gene in Latin America (Tijet et al., 2017), to adapt to the XDR phenotype (Magiorakos et al., 2012) by conjugation and/or transformation assays. The conservation of ARG in the evolved MDR and XDR-*E. coli* M19736 strains showed different patterns of maintenance as well as variability in the capacity to transfer the acquired conjugative plasmids including the co-transfer of different plasmids in several combinations. In addition, we identified EVs in the native and in the evolved XDR-*E. coli* M19736 strains with crucial content for the competence of AMR in the nosocomial niche, including the *bla*_CTX-M-15_ gene though the plasmid containing this gene, pDCAG1-CTX-M-15, was previously lost. Collectively, this scenario reveals the relevant role of sporadic clones as common vectors for the dissemination of acquired AMR within the framework of the HGT processes.

## 2 Methods

### 2.1 Bacterial strains and growth conditions

*Escherichia coli* M19736 was isolated in November 2015, in Argentina, from the blood of a patient with peritonitis secondary to colon cancer (Tijet et al., 2017). The strain was shown by MLST to be a single-locus variant of ST615 (Rapoport et al., 2016; Tijet et al., 2017). *Escherichia coli* M19736 has the pM19736 plasmid with the size of 63,230 bp that harbors an *mcr-1* gene as described previously (Tijet et al., 2017). *Escherichia coli* SM5 (Sennati et al., 2012), *Klebsiella pneumoniae* HA7Kp (Knecht et al., 2022), *Klebsiella pneumoniae* HA31Kp (Álvarez et al., 2024), and *Serratia marcescens* SM938 (this study) (**Table 1**) were used as donors for conjugation assays as described below. Also, *E. coli* J53 was used as a laboratory model for the control of the conjugation experiments.

**Table 1:** Bacterial strains used for the experiments.

Strain	ST	Year of isolation	Origin of the sample	Inc. groups	Sequencing technique	Antibiotic susceptibility^a^	Reference
*Escherichia coli* M19736	615	2015	Blood culture	IncI2, IncFII, IncI1-I (Alpha)	Illumina MiSeq	**Resistant**: AMN, AMC, CMP, CIP, FOS, TET **Intermediate**: AMS	Rapoport et al. (2016); Tijet et al. (2017)
*Escherichia coli* SM5	131	2010	Urine	IncFIB, IncFII	Illumina MiSeq	**Resistant**: AMN, CAZ, CRO, FEP, AZT, TAZ, AMC, AMS, CIP, TMS, FOS **Intermediate**: AKN	Sennati et al. (2012)
*Klebsiella pneumoniae* HA7Kp	18	2018	Rectal swab	IncM1, IncHI1B/IncFIB	Illumina MiSeq	**Resistant**: AMN, CAZ, CRO, AZT, MEM, IMI, TAZ, AMC, AMS, TMS **Intermediate**: FEP, TET	Knecht et al. (2022)
*Klebsiella pneumoniae* HA31Kp	11	2018	Tracheal aspirate	IncFII, Col440I, IncFIB(K)	Illumina MiSeq	**Resistant**: AKN, GEN, AMN, CAZ, CRO, FEP, AZT, MEM, IMI, TAZ, AMC, AMS, CMP, CIP, TMS, FOS	Álvarez et al. (2024)
*Serratia marcescens* SM938	NA	2018	Blood culture	IncC	Illumina MiSeq/MinION	**Resistant**: AMN, CAZ, CRO, TAZ, AMC, AMS, MEM, IMI **Intermediate**: FEP, AZT	This study
*Escherichia coli* TOP 10::paadB	10	NA	Laboratory	p15A	Illumina MiSeq	**Resistant**: GEN, CMP	Quiroga (2012)
*Escherichia coli* J53	10	NA	Laboratory model	NA	Illumina NextSeq 500/MinION	**Resistant**: AZI	Matsumura et al. (2018)

General information on the bacterial strains used for the experiments including sequence type (ST), source of isolation, year of isolation, sequencing technique, incompatibility groups (inc. groups), and antibiotic susceptibility.

AKN, amikacin; GEN, gentamicin; AMN, ampicillin; CAZ, ceftazidime; CRO, ceftriaxone; FEP, cefepime; AZT, aztreonam; MEM, meropenem; IMI, imipenem; TAZ, piperacillin/tazobactam; AMC, amoxicillin/clavulanic; AMS, ampicillin/sulbactam; CMP, chloramphenicol; CIP, ciprofloxacin; TMS, trimethoprim/sulfamethoxazole; FOS, fosfomycin; TET, tetracycline; AZI, sodium azide; NA, not applicable.

a The results were interpreted according to the Clinical and Laboratory Standards Institute guidelines (CLSI, 2023).

The plasmid paadB from the *E. coli* TOP10::paadB strain was used for the transformation assays (**Table 1**). The *aadB* gene cassette from *S. marcescens* SCH909 (Gambino et al., 2021) was subcloned from a pCR2.1TOPO vector (Invitrogen, Carlsbad, CA) into the commercial vector pACYC184; paadB is resistant to chloramphenicol due to the background of its vector and to gentamicin due to the *Pc* promoter contained upstream the *aadB* gene cassette (Gambino et al., 2021).

All the strains were grown in Luria–Bertani (LB) broth at 37°C with shaking (150 rpm). When needed, antibiotics were used at the following concentrations: ceftazidime (8 μ/ml), meropenem (2 μg/ml), or gentamicin (25 μg/ml).

### 2.2 Antibiotic susceptibility testing, minimum inhibitory concentration, and phenotypic detection of β-lactamases

Receptor, donor, and transconjugant strains were tested by antibiotic susceptibility testing (AST) and minimum inhibitory concentration (MIC). AST was carried out by the agar disk diffusion method; MIC was determined by agar dilution. Both assays were conducted according to the Clinical and Laboratory Standards Institute (CLSI) guidelines (CLSI, 2023), and the results were interpreted under the same guidelines. On the other hand, phenotypic detection of β-lactamases was determined by two tests. First, the detection of carbapenemases was performed by the modified Hodge test as previously described (Pasteran et al., 2016). Secondly, the synergy produced between the extended-spectrum cephalosporins and clavulanic acid was used for the detection of extended-spectrum β-lactamase.

### 2.3 Polymerase chain reaction assays

Total DNA extraction was done using the boiling technique for all the experiments of conjugation and transformation. All polymerase chain reaction (PCR) reactions were done using 2 U of Taq DNA polymerase (Inbio Highway, Tandil, Argentina) in 0,5× Taq buffer (Inbio Highway) supplemented with 2,5 mM of MgCl_2_, 0,2 mM dNTP mix, and 0,4 uM of each primer in a final volume of 25 µl The PCR conditions were 5 min at 95°C, 30 cycles of 30 s at 95°C, 45 s at the appropriate annealing temperature and 1 min at 72°C, followed by a final extension of 5 min at 72°C.

Detection of each gene of interest—*bla*_CTX-M-15_, *bla*_KPC-2_, *bla*_NDM-5_, *bla*_NDM-1_, *aadB*, and/or *mcr-1* genes—was conducted with specific primers listed in **Table 2**. PCR products were separated on agarose gels by electrophoresis, stained with SYBR green, and visualized by UV transillumination.

**Table 2:** Primers used for the detection of genes of interest.

Gene/plasmid of interest	Primers	Sequence (5′–3′)	Expected size (bp)	Reference
***mcr-1***	ForMCR-1	AGTCCGTTTGTTCTTGTGGC	320	Rebelo et al. (2018)
	RevMCR-1	AGATCCTTGGTCTCGGCTTG		
***bla*_CTX-M-15_**	CTX-M-15F	CGTCACGCTGTTGTTAGGAA	612	This study
	CTX-M-15R	CGGTGGTATTGCCTTTCATC		
***bla*_KPC-2_**	KPC-F	CCGTCAGTTCTGCTGTC	916	Ramírez et al. (2013)
	KPC-R	CGTTGTCATCCTCGTTAG		
***bla*_NDM_**	NDM1-F	CGCGAAGCTGAGCACCGCATTAG	733	This study
	NDM1-R	CTATCGGGGGCGGAATGG		
***aadB***	SULPRO3	GCCTGACGATGCGTGGA	623	Gambino et al. (2021)
	aadBR5′	AAGAATCCATAGTCCAACTCC		
**pACYC184**	PACYC1845′	TGTAGCACCTGAAGTCAGCC	496	Gravel et al. (1998)
	PACYC1843′N	GTGATGTCGGCGATATAGGC		

### 2.4 Conjugation assays

Conjugation experiments were performed according to a method described previously (Di Noto et al., 2016). Briefly, mating assays were carried out on LB agar plates. *Escherichia coli* SM5 (Caz^R^), HA7Kp (Mem^R^), *K. pneumoniae* HA31Kp (Mem^R^), and *S. marcescens* SM98 (Mem^R^) were used as donor strains, while *E. coli* M19736 (Cmp^R^) and J53 (Azi^R^) were used as recipient strains. Donor and recipient strains were diluted from saturated overnight cultures into 12 ml and grown until OD_600 nm_ ~0.6 at 37°C. The cells were harvested by centrifugation, mixed together in a ratio of 1:1, and spotted onto LB plates. They were also spotted individually on LB plates as controls. After 18 h of incubation at 37°C, mating spots were washed and resuspended in saline; serial dilutions were plated onto LB agar with the specific antibiotic to select for donor, recipient, or transconjugant cells (meropenem 3 µg/ml or ceftazidime 8 µg/ml and chloramphenicol 50 µg/ml or sodium azide 150 µg/ml). Conjugation frequency was expressed as the number of transconjugant cells per donor cell in the mating mixture at the time of plating. Transconjugants obtained were checked by plating on CROMagar and LB plates with double antibiotics. Then, the different genes of interest (*bla*_CTX-M-15_, *bla*_KPC-2_, *bla*_NDM-5_, and *bla*_NDM-1_) were detected by PCR for each transconjugant. The original recipient strains [*E. coli* M19736 (Cmp^R^) and J53 (Azi^R^)] were used as negative controls, and the donor strains [*E. coli* SM5 (Caz^R^), HA7Kp (Mem^R^), *K. pneumoniae* HA31Kp (Mem^R^), and *S. marcescens* SM98 (Mem^R^)] were used as positive controls (**Table 1**).

### 2.5 Transformation assays

*Escherichia coli* M19736 was treated with 10% glycerol previously. Afterward, electroporation was performed with a Gene Pulser™ apparatus (Bio-Rad Laboratories, Denver, USA) and conducted using the following parameters: 200 Ω resistance, 25 mF capacitance, and 2 kV voltage, resulting in a time constant between 4.5 and 5.0 ms. Transformed *E. coli* cells were recovered in a 1-ml LB broth and incubated at 37°C with a 200-rpm shaking for 2 h before being plated on LB agar plates supplied with gentamicin (25 μg/ml) using 100 µl.

### 2.6 Plasmid maintenance assay

Each strain of interest was cultured in 5 ml of LB broth without antibiotic pressure and incubated at 37°C ON with shaking (200 rpm). Consecutive subcultures were made for 10 days. An aliquot was taken from each experiment on the 1st and 10th days and plated on LB agar without antibiotics. From each of the replicates, 30 colonies were selected and analyzed. DNA was then extracted from each of these colonies using the boiling method. Next, each of the 30 colonies taken per replicate was tested by PCR for the presence or absence of each of the acquired genes of interest (*bla*_CTX-M-15_, *bla*_KPC-2_, *bla*_NDM-1_, *bla*_NDM-5_, *mcr-1* and *aadb*). The maintenance percentage of each replicate was then calculated as follows: $\%Maintenance=[\frac{\left(n_{pc}+100\%\right)}{n_{t}}]$ , where *n_pc_* is the number of positive colonies for the gene of interest and *n_t_* is the total number of colonies tested (*n_t_* = 30). Then, the average $\left(\bar{X}\right)$ of three replicates performed was calculated with their respective standard deviations (SDs).

### 2.7 Isolation and purification of EV

EVs were isolated from the late log-phase (16 h) culture of *E. coli* M19736 and evolved XDR-*E. coli* M19736 on day 1 of subculture. In brief, cells were cultivated in 600 ml of LB broth with 10 µg/ml of ceftazidime and subinhibitory concentrations of meropenem ~14 h at 37°C. The next day, the cultures were adjusted to OD_600_ ~0.7. The cells were pelleted by centrifugation (9,500 rpm, 4°C for 20 min), and the supernatant was filtered through a 0.22-μm membrane filter (Merck Millipore, Tullagreen, Carrigtwohill, Co. Cork, Ireland) to remove cells and cellular debris. The filtrate was subjected to ultracentrifugation (100,000*g*) for 2 h at 4°C using a P45AT(RP45T) fixed angle rotor (HIMAC CP80NX). For washing the EV, the pellet suspended in EV buffer (137 mM of NaCl and 20 mM of HEPES [pH 7.5]) was ultracentrifuged (100,000*g*) for 1 h at 4°C using the same rotor. The pellet was finally resuspended in 1500 µl of buffer, filtered and frozen at −80°C. The EVs were grown in 2 ml of LB broth to test for any bacterial growth.

### 2.8 Dynamic light scattering

The hydrodynamic diameter (Dh) and the size distribution (polydispersity index, PDI) of different EV sources were assayed by dynamic light scattering (DLS) (DLS, Zetasizer Nano-ZS, Malvern Instruments) at a scattering angle of 173°. The nano-ZS contains a 4-mW He–Ne laser operating at a wavelength of 633 nm, a digital correlator ZEN3600, and non-invasive backscatter (NIBS^®^) technology. For the measurement, 200 μl of vesicles suspended in EV buffer (137 mM NaCl and 20 mM HEPES [pH 7.5]) were used. All the samples were analyzed at 25°C. Viscosities were between 0.8880 and 0.8872 cP. Results were expressed as mean ± standard deviation (SD) of three independent samples prepared in identical conditions. Data for each single specimen were the result of at least six runs.

### 2.9 Transmission electron microscopy

To verify the presence of intact EV, the preparations were analyzed using transmission electron microscopy (TEM). The EV suspension was fixed in nickel grids for TEM with carbon (200 mesh) (Agar Scientific Ltd., Stansted, Essex, UK), with 2% glutaraldehyde, 4% formaldehyde, and 5% sucrose in PBS; washed three times with ultrapure water; stained with 3% uranyl acetate; allowed to dry for at least 30 min; and examined under a transmission electron microscope (Zeiss EM 109T equipped with Gatan ES1000W digital camera).

### 2.10 Mass spectrometry analysis

EV proteins were quantified by the Micro BCA™ Protein Assay Kit (Thermo Fisher Scientific, Rockford, IL, USA). Protein digestion from lysed EV of native *E. coli* M19736 was performed. We used 40 μg of protein from EV based on the Micro BCA results. The proteins were reduced and alkylated with 10 mM of DTT and 20 mM of iodoacetamide and then precipitated with 15% trichloroacetic acid/acetone and processed for liquid chromatography–MS/MS (LC-MS/MS) analysis. Mass spectrometry analysis was performed at the Proteomics Core Facility (CEQUIBIEM), University of Buenos Aires/CONICET (National Research Council) by analyzing the digests by nanoLC-MS/MS in a Thermo Scientific Q Exactive Mass Spectrometer coupled to a nanoHPLC EASY-nLC 1000 (Thermo Scientific). For the LC-MS/MS analysis, approximately 2 μg of peptides were loaded onto the column and eluted for 120 min using the reverse phase column (C18, 2 µm, 100 A, 50 µm × 150 mm) EASY-Spray Column PepMap RSLC (P/N ES801) suitable for separating protein complexes with a high degree of resolution. The flow rate used for the nano column was 300 nl min^−1^, and the solvent ranged from 7% B (5 min) to 35% (120 min). Solvent A was 0.1% formic acid in water, whereas solvent B was 0.1% formic acid in acetonitrile. The injection volume was 2 µl. The MS equipment has a high collision dissociation cell (HCD) for fragmentation and an Orbitrap analyzer (Thermo Scientific, Q-Exactive). A voltage of 3.5 kV was used for electrospray ionization (Thermo Scientific, EASY-Spray).

XCalibur 3.0.63 (Thermo Scientific) software was used for data acquisition and equipment configuration that allows peptide identification and chromatographic separation. Full-scan mass spectra were acquired in the Orbitrap analyzer. The scanned mass range was 400–1,800 *m*/*z*, at a resolution of 70,000 at 400 *m*/*z*, and the 12 most intense ions in each cycle were sequentially isolated, fragmented by HCD, and measured in the Orbitrap analyzer. Peptides with a charge of +1 or with an unassigned charge state were excluded from fragmentation for MS2.

#### 2.10.1 Analysis of mass spectrometry data

Q Exactive raw data were processed using Proteome Discoverer software (version 2.1.1.21 Thermo Scientific) and searched against the *E. coli* sequence database with trypsin specificity and a maximum of 1 missed cleavage per peptide. Carbamidomethylation of cysteine residues was set as a fixed modification, and oxidation of methionine was set as a variable modification. Proteome Discoverer searches were performed with a precursor mass tolerance of 10 ppm and product ion tolerance of 0.05 Da. Protein hits were filtered for high-confidence peptide matches with a maximum protein and peptide false discovery rate of 1% calculated by employing a reverse database strategy.

### 2.11 EV protein analysis

The localization of proteins was mostly acquired using DAVID (https://david.ncifcrf.gov/home.jsp). The biological process of EV proteins was derived from Gene Ontology, UniProt, and KEGG (https://www.genome.jp/kegg/). Proteins associated with antibiotic response/resistance were predicted using DAVID.

#### 2.11.1 Protein–protein interaction network analysis

Protein–protein interaction (PPI) data were downloaded using the STRING v10.5 database (Szklarczyk et al., 2017). A PPI network of EV proteins from STRING was incorporated in Cytoscape 3.9.0 (Shannon et al., 2003), and using the Cytoscape StringApp (Doncheva et al., 2019), we constructed, analyzed, and visualized the PPI network. Gene Ontology and KEEG enrichment analysis was performed for the EV proteins.

### 2.12 Determination of DNA in EV

Intravesicular DNA was quantified following the method of Rumbo et al. (2011) with a few modifications. Fifty micrograms of EVs were treated with DNAse RQ1-Free RNAse 1U/µl (Promega) at 37°C for 30 min to hydrolyze the free and surface-associated DNA. The reaction was stopped with a stop solution and incubation was conducted at 65°C for 10 min. DNase-treated EVs were then lysed with 0.125% Triton X-100 (Sigma-Aldrich, USA) solution for 30 min at 37°C, and DNA was purified using a QIAamp DNA Mini Kit with the protocol for crude cell lysates and other samples (Qiagen, Maryland, USA), according to the manufacturer’s instructions. The DNA was quantified using the Nano-500 Micro-spectrophotometer. The purified DNA was used for further PCR using the primers listed in **Table 2**.

### 2.13 DNA extraction, DNA sequencing, and sequence assembly

DNA of *E. coli* M19736 and evolved XDR-*E. coli* M19736 on day 1 of subculture was extracted using the mini kit QIAamp DNA (Qiagen) following the manufacturer’s protocol for Gram-negative bacteria. The concentration and purity were measured using a NanoDrop instrument (Nano-500 Micro-Spectrophotometer, Allsheng, Hangzhou, China).

DNA was sequenced on a MiSeq sequencer (Illumina pair ends). The sequencing was performed in the Genomics and Bioinformatics Unit of ANLIS Malbrán (Argentina), and the library preparation was made according to the manufacturer’s protocol. Read quality metrics were evaluated using FASTQC v0.11.9. To remove the low-quality reads and the remaining adapters from the sequencing, trimmomatic v0.39 was used. The parameters used were as follows: -threads 8 -phred33 ILLUMINACLIP:TruSeq3.fa:2:30:10 TRAILING:20 SLIDINGWINDOW:4:20 MINLEN:50. The trimmed Fastq files were evaluated using fastqc to determine their quality. Finally, we performed a short-read assembly using Unicycler v0.4.8 (Wick et al., 2017) with default options. Unicycler was executed by the command line in the GNU/Linux environment. Subsequently, the quality of the assembled files was evaluated using the QUAST v5.0.2 program, using default parameters.

### 2.14 Genomic and plasmid analysis

Consensus sequences of the complete assembly were imported into the RAST (Aziz et al., 2008) and PROKKA (Seemann, 2014) databases. The search for all ARGs and efflux pumps associated with AMR was performed using ResFinder (Zankari et al., 2012), RGI 6.0.2, and CARD 3.2.7 online databases with a minimum identity of 95%. In addition, chromosomal mutations associated with AMR were searched using PointFinder available in the ResFinder database. PlasmidFinder v 2.1.6 (Clausen et al., 2018; Carattoli and Hasman, 2020) was used to detect the plasmids in our samples using the default parameters. VRprofile2 v2.0 was used to predict mobilome (Wang et al., 2022). Conjugation systems were searched against the NCBI database, and OriT was detected using the online tool oriTfinder (Li et al., 2018). The search and detection of toxin–antitoxin systems was performed using the online tool TADB v2.0 using the default parameters (Guan et al., 2023).

### 2.15 Statistical analysis

Statistical analysis of the data obtained was performed using the GraphPad Prism 8.0.2 program (GraphPad, La Jolla, CA, USA). Variables were expressed as median (interquartile range, IQR) and compared by Kruskal–Wallis followed by Dunn’s *post-hoc* test. We looked for statistically significant differences between conjugation frequencies of the *E. coli* M19736 strain and the laboratory control strain *E. coli* J53. *p*-values<0.05 were regarded as statistically significant.

## 3 Results

### 3.1 Ability of *Escherichia coli* M19736 to acquire plasmids from different species

**Figure 1:**
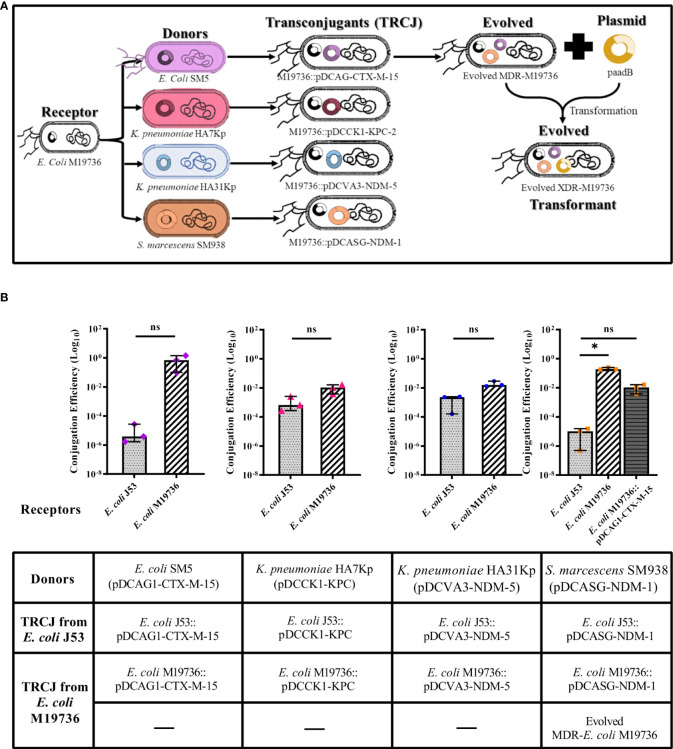
Conjugation/transformation assays of different multidrug-resistant plasmids with *Escherichia coli* M19736 and *E. coli* J53 as laboratory control. Panel **(A)** shows the acquisition of the different plasmids by conjugation or transformation of *E. coli* M19736. Each color represents the plasmids that were transferred: pDCAG1-CTX-M-15 (violet), pDCCK1-KPC (pink), pDCVA3-NDM-5 (blue), pDCASG-NDM-1 (orange), and paadB (yellow). Five transconjugants using *E. coli* M19736 as receptor (*E. coli* M19736::pDCAG1-CTX-M-15, *E. coli* M19736::pDCCK1-KPC, *E. coli* M19736:: pDCVA3-NDM-5, *E. coli* M19736::pDCASG-NDM-1, and evolved MDR-*E. coli* M19736) and transformant XDR-*E. coli* M19736 were generated. Panel **(B)** shows each donor and transconjugant (TRCJ) obtained in the different experiments using *E. coli* M19736 and *E. coli* J53 as receptors. *Escherichia coli* J53 as control is depicted as light gray with dotted bars, *E. coli* M19736 as black diagonal line bars, and evolved MDR-*E. coli* M19736 as dark gray with horizontal line bars. Data show the comparison of conjugation efficiencies of each plasmid between *E. coli* M19736 and *E. coli* J53. Conjugation efficiencies were calculated as the quotient between the number of transconjugants (Tc) and the number of donors (D) (Tc/D) in triplicates. Data show the median with range from three independent experiments (*n* = 3). Significant differences between groups are indicated: *p< 0.05; ns, not significant.

*Escherichia coli* M19736 was tested as a receptor to receive crucial ARG by transformation and conjugation using different relevant plasmids as donors (**Table 3**) including i) pDCAG1-CTX-M-15 (>112.000 bp, IncFII) from the clinical strain *E. coli* SM5 that harbors *bla*_CTX-M-15_, ii) pDCCK_1_-KPC (>77.218 bp, IncM1) from the clinical strain *K. pneumoniae* HA7Kp that harbors *bla*_KPC-2_, iii) pDCVA3-NDM-5 (>534.520 bp, IncFII) from the clinical strain *K. pneumoniae* HA31Kp that harbors *bla*_NDM-5_, iv) pDCASG-NDM-1 (137.269 bp, IncC) from the clinical strain *S. marcescens* SM938 that harbors *bla*_NDM-1_, and v) recombinant plasmid paadB from *Escherichia coli* TOP 10::paadB (5.877 bp, p15A) that harbors the *aadB* gene cassette. We identified by bioinformatics analysis that the four clinical plasmids had conjugation systems (**Table 3**), all of them being conjugative to *E. coli* J53 and *E. coli* M19736 in our experimental conditions (**Figure 1**). Also, *E. coli* M19736 was able to acquire paadB by chemical transformation (**Figure 1A**). Conjugation was performed as previously described, showing that *E. coli* M19736 was able to acquire the four plasmids (**Figure 1B**). Although all conjugation efficiencies were higher when *E. coli* M19736 was the receptor strain, a statistical difference between *E. coli* M19736 and *E. coli* J53 was only found when *S. marcescens* SM938 (pDCASG6-NDM-1) was used as a donor (**Figure 1B**).

**Table 3:** Features of plasmids used for the experiments.

Plasmid	Bacterial strain	Plasmid or pseudomolecule size	Inc group^a^	Antibiotic resistance genes of the plasmids^b^	Conjugation genes	Toxin/antitoxin system	GenBank accession no.^c^
**pM19736-MCR-1**	*Escherichia coli* M19736	63,230 bp	IncI2	*mcr-1.1*	*pilVUTSRQPON/traKJIHG/traEDCB/pilML/trbJL*	*relE/B* and *TsxA/B*	FR851304 and JN983044
**pDCAG1-CTX-M-15**	*Escherichia coli* SM5	>112.000 bp	IncFII	*tet(B)*, *catB3*, *dfrA8*, *sul2*, *bla* _CTX-M-15_ , *bla*_TEM-1B_, *bla*_OXA-1_, *aac(6′)-Ib-cr*, *aph(6)-Id*, *aph(3″)-Ib*	*FinO/traXIDTSGH/trbFJB/traQ/trbA/traF/trbE/traN/trbC/traUW/trbI/traCRV/trbD/traPBKELAJM*	*CcdA/B* and *pemK/L*	AY458016.1
**pDCCK1-KPC**	*Klebsiella pneumoniae* HA7Kp	>77.218 bp	IncM1	*bla* _KPC-2_	*traHIJK/traLMNOPQRUWXY/trbCBAN*	*pemK/L*	AF550415.2
**pDCVA3-NDM-5**	*Klebsiella pneumoniae* HA31Kp	>534.520 bp	IncFII	*fosA*, *catB3*, *sul1*, *aac(6′)-Ib-cr*, *oqxA*, *qnrS1*, *oqxB*, *erm(B)*, *mph(A)*, *bla*_CTX-M-15_, *bla*_TEM-1B_, *bla*_OXA-1_, *bla* _NDM-5_ , *qacE, aadA2*, *aac(6′)-Ib-cr*, *aph(3′)-Ia*, *dfrA12*, *rmtB*	*FinO/traXID/traGH/trbFJB/traQ/trbA/traF/*/*traN/trbC/traUW/trbI/traCRV/trbD/traPBKELAJM*	*AAA-ATPase/relB*, *pemK/L*, and *HipA/B*	AY458016.1
**pDCASG-NDM-1**	*Serratia marcescens* SM938	137.269 bp	IncC	*bla* _NDM-1_ , *ble*_MBL_, *bla*_CMY-6_, *qacEΔ1, sul1*, *Δbla*_OXA-1_/*aac(6′)-Ib3*	*traFHG/traID traLEKBVA/traC/trhF*/*traWUN*	*HigA/B*	JX141473.1
**paadB**	*Escherichia coli* TOP 10::paadB	5.877 bp	p15A	*aadB*, *catA1*	–	–	Not applicable

The results found by bioinformatics analysis of the plasmids used as donors in the transformation and conjugation assays.

a The replicon shown for each strain corresponded to the genetic location where the carbapenemase gene, the bla_CTX-M-15_, or the *aadB* gene cassette was found.

b Underlined genes were used as conjugation/transformation biomarkers.

c GenBank accession number of plasmids used as reference.

ARG acquisition by *E. coli* M19736 and *E. coli* J53 as receptor strains was verified by PCR of conjugation markers (**Table 2**), phenotypic detection of β-lactamases, AST (**Supplementary Table 1**), and MIC (**Table 4**). In the experiments of successive conjugation assays, the evolved MDR-*E. coli* M19736 was able to harbor simultaneously *mcr-1*, *bla*_CTX-M-15_, and *bla*_NDM-1_ genes (**Figure 1A**). The evolved XDR-*E. coli* M19736 was able to acquire also the *aadB* gene later by transformation assay (**Figure 1A**). Susceptibility tests showed that the evolved XDR-*E. coli* M19736 became resistant to all antibiotics tested except to trimethoprim/sulfamethoxazole (**Supplementary Table 1**). The MIC for carbapenem antibiotics (ERT and MEM) was slightly higher in *E. coli* M19736::pDCVA3-NDM-5, evolved MDR-*E. coli* M19736, and evolved XDR-*E.coli* M19736 than the respective donor strains and transconjugants of *E. coli* J53 (**Table 4**).

**Table 4:** Minimum inhibitory concentration (MIC) of *Escherichia coli* strains.

	CAZ	ERT	MEM	GEN
***E. coli* ATCC 25922**	0.5 (S)	0.016 (S)	0.03 (S)	0.5 (S)
***E. coli* M19736**	0.03 (S)	0.5 (S)	0.25 (S)	0.03 (S)
***E. coli* J53**	0.25 (S)	0.08 (S)	0.03 (S)	0.5 (S)
***E. coli* SM5**	64 (R)	0.12 (S)	0.06 (S)	0.5 (S)
***E. coli* M19736::pDCAG1-CTX-M-15**	>64 (R)	0.5 (S)	0.25 (S)	0.03 (S)
***E. coli* J53::pDCAG1-CTX-M-15**	64 (R)	0.08 (S)	0.03 (S)	0.5 (S)
***K pneumoniae* HA7Kp**	32 (R)	8 (R)	8 (R)	0.25 (S)
***E. coli* M19736::pDCCK1-KPC**	32 (R)	4 (R)	2 (I)	0.03 (S)
***E. coli* J53::pDCCK1-KPC**	16 (R)	2 (R)	4 (R)	0.5 (S)
***K. pneumoniae* HA31Kp**	>64 (R)	32 (R)	32 (R)	>64 (R)
***E. coli* M19736::pDCVA3-NDM-5**	>64 (R)	64 (R)	32 (R)	0.03 (S)
***E. coli* J53::pDCVA3-NDM-5**	>64 (R)	2 (R)	4 (R)	0.5 (S)
***S. marcescens* SM938**	>64 (R)	8 (R)	8 (R)	2 (S)
***E. coli* M19736::pDCASG-NDM-1**	>64 (R)	16 (R)	8 (R)	0.03 (S)
***E. coli* J53::pDCASG-NDM-1**	>64 (R)	32 (R)	16 (R)	0.5 (S)
**Evolved MDR*-E. coli* M19736**	>64 (R)	32 (R)	16 (R)	0.03 (S)
**Evolved XDR-*E. coli* M19736**	>64 (R)	32 (R)	16 (R)	32 (R)

Results were interpreted according to the Clinical and Laboratory Standards Institute guidelines (CLSI, 2023).

CAZ, ceftazidime; ERT, ertapenem; MEM, meropenem; GEN, gentamicin. Interpretation results: I, intermediate; R, resistance; S, susceptible.

### 3.2 Maintenance of plasmids in native and evolved MDR and XDR-*Escherichia coli* M19736 strains

**Figure 2:**
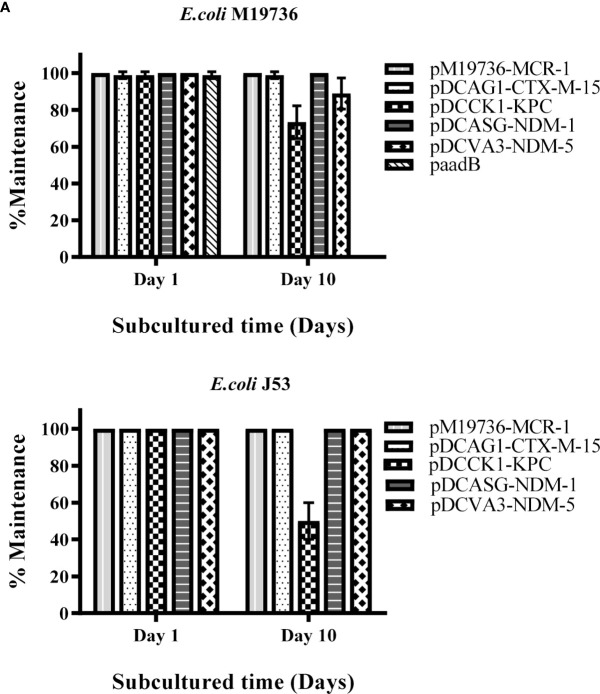
Maintenance of crucial ARG harbored by plasmids. Each color/pattern shows the percentage of maintenance of each ARG that was acquired in its respective plasmid by *E. coli* M19736 **(A)** or by *E. coli* J53 **(B)**. The *mcr-1* gene in pM19736-MCR-1, the *bla*_CTX-M-15_ gene in pDCAG1-CTX-M-15, the *bla*_KPC-2_ gene in pDCCK_1_-KPC, the *bla*_NDM-5_ gene in pDCVA3-NDM-5, the *bla*_NDM-1_ gene in pDCASG-NDM-1, and the *aadB* gene in paadB were used as target for PCR detection with primers from **Table 2**, respectively. Data shows the mean ± SD from three independent experiments performed in triplicate (*n* = 3).

Firstly, we evaluated the maintenance of plasmids of each donor cell (*Escherichia coli* SM5, *K. pneumoniae* HA7Kp, *K. pneumoniae* HA31Kp, and *S. marcescens* SM938) on the 1st and 10th days after being subcultured without antibiotic pressure at 37°C. All plasmids were maintained at 100% of each assay (data not shown). Then, each transconjugant of *E. coli* M19736, or transformant in the case of XDR-*E. coli* M19736 (**Figure 1**), was evaluated for its ability to maintain ARG (*bla*_CTX-M-15_, *bla*_KPC-2_, *bla*_NDM-5_, *bla*_NDM-1_, or *aadB*) by doing the same assay without antibiotic pressure at 37°C (**Figure 2**). At the same time, the maintenance of the *mcr-1* gene was evaluated in all combinations. Each one of the four clinical plasmids (pDCAG1-CTX-M-15, pDCCK1-KPC, pDCVA3-NDM-5, and pDCASG6-NDM-1) harbored a different toxin/antitoxin system (**Table 3**). The transconjugants were able to maintain each gene of interest on the first day of subculture at 100%. Each transconjugant or transformant maintained *bla*_CTX-M-15_ (pDCAG1-CTX-M-15), *bla*_KPC-2_ (pDCCK1-KPC), *bla*_NDM-5_ (pDCVA3-NDM-5), *bla*_NDM-1_ (pDCASG6-NDM-1), and *aadB* (paadB) at 98.9%, 73.3%, 88.9%, 100%, and 0%, respectively, on the 10th day of subculture (**Figure 2**).

**Figure 3:**
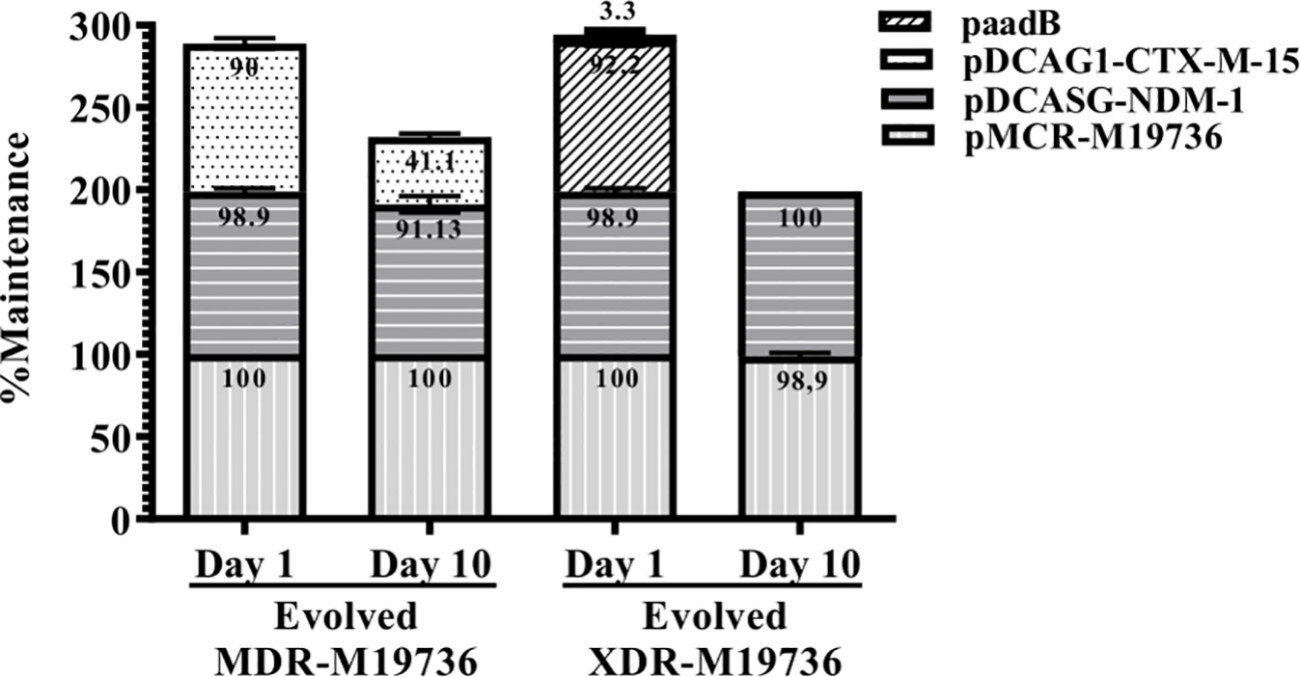
Maintenance of the ARG-plasmid located in the evolved MDR and XDR *E. coli* M19736 strains. The bars represent an evolved MDR-*E. coli* M19736 or evolved XDR-*E. coli* M19736 on the 1st day and 10th day of subculture. Each color/pattern and number within each bar represents the percentage of maintenance for the conjugated plasmids. Data shown are the mean ± SD from three independent experiments performed in triplicate (*n* = 3).

Interestingly, when the evolved MDR and XDR-*E. coli* M19736 strains acquired progressively *bla*_CTX-M-15_ (pDCAG1-CTX-M-15) and *bla*_NDM-1_ (pDCASG6-NDM-1) or acquired *bla*_CTX-M-15_ (pDCAG1-CTX-M-15), *bla*_NDM-1_ (pDCASG6-NDM-1), and *aadB* (paadB) plasmids, respectively, a different pattern of maintenance was found (**Figure 3**). In the case of evolved MDR-*E. coli* M19736, pDCAG1-CTX-M-15 and pDCASG-NDM-1 were maintained at 41.1% and 91.1%, respectively, on the 10th day of subculture (**Figure 3**). When the evolved XDR-*E. coli* M19736 that harbored *bla*_CTX-M-15_ (pDCAG1-CTX-M-15), *bla*_NDM-1_ (pDCASG6-NDM-1), and *aadB* (paadB) genes was tested, we found that *bla*_CTX-M-15_ (pDCAG1-CTX-M-15) and *aadB* (paadB) genes were lost while maintaining the *bla*_NDM-1_ gene (pDCASG6-NDM-1) at 98.9% on the 10th day of subculture. Remarkably, in all cases without antibiotic pressure, *E. coli* M19736 maintained the *mcr-1* gene.

On the other hand, bioinformatics analysis of the WGS of the evolved XDR-*E. coli* M19736 from subculture on day 1 confirmed the presence of eight ARGs found in pM19736-MCR-1 and pIncFII-M19736 plasmids and chromosome from the host *E. coli* M19736 strain, five ARGs from pDCASG-NDM-1 as expected, and two ARGs from paadB (**Table 5**). The replication origins of these plasmids were also found. In contrast, no antibiotic determinant or replication origins of the pDCAG1-CTX-M-15 plasmid were found in the evolved XDR-*E. coli* M19736 which could be due to the fact that pDCAG1-CTX-M-15 was rapidly lost on day 1 as shown in our maintenance experiments (see below) (**Figure 3**).

**Table 5:** ARGs found in the genome of the evolved XDR-*E. coli* M19736.

Evolved XDR-*E. coli* M19736	*E. coli* M19736	pIncFII-M19736	pM19736-MCR-1	pDCASG6-NDM-1	paadB
*fosL1*	*fosL1*	*aph(6)-Id*	*mcr-1.1*	*aac(6′)-Ib3*	*aadB*
*tet(B)*	*tet(B)*	*aph(3″)-Ib*		*bla*_CMY-6_	*catA1*
*aph(6)-Id*		*bla*_TEM-1B_		*bla*_NDM-1_	
*aph(3″)-Ib*		*floR*		*sul1*	
*bla*_TEM-1B_		*sul2*		*qacE*	
*floR*					
*sul2*					
*mcr-1.1*					
*aac(6′)-Ib3*					
*bla*_CMY-6_					
*bla*_NDM-1_					
*sul1*					
*qacE*					
*aadB*					
*catA1*					

The data show the results found by ResFinder of ARGs identified in the genome of the evolved XDR-*E. coli* M19736, *E. coli* M19736, and plasmids of interest.

### 3.3 Ability of the evolved MDR and XDR-*Escherichia coli* M19736 strains to disseminate the acquired conjugative plasmids

**Figure 4:**
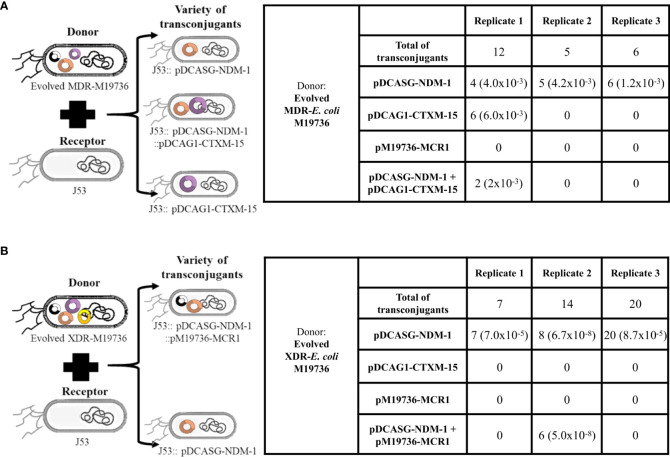
Dissemination of plasmids co-infecting evolved *Escherichia coli* M19736 strains to *E. coli* J53. Evolved MDR-*E. coli* M19736 and XDR-*E. coli* M19736 were tested as donors of co-infecting clinical conjugative plasmids using as receptor *E. coli* J53 **(A, B)**. The selection was performed with 8 µg/ml of ceftazidime. The number of colonies detected with each gene (*bla*_NDM-1_, *bla*_CTX-M-15_, and/or *mcr-1*) in transconjugants is shown. Numbers in parentheses represent the conjugation efficiencies of each experiment for the plasmids. Conjugation efficiencies were calculated as the quotient between the number of transconjugants (Tc) and the number of donors (D) (Tc/D). Both evolved strains were able to transfer the *bla*_NDM-1_ gene located in pDCASG-NDM-1 to *E. coli* J53 from three independent biological replicates. When the evolved MDR-*E. coli* M19736 was used as donor, two other genotypes were identified in transconjugants in one replicate **(A)**. The first genotype harbored simultaneously plasmids of the *bla*_NDM-1_ (pDCASG-NDM-1) and *bla*_CTX-M-15_ (pDCAG1-CTX-M-15) genes in *E. coli* J53 transconjugants. The second one harbored only the *bla*_CTX-M-15_ (pDCAG1-CTX-M-15) gene. When the evolved XDR-*E. coli* M19736 was used as donor, in one replicate, another genotype was identified in which the *bla*_NDM-1_ (pDCASG-NDM-1) and *mcr-1* (pM19736-MCR-1) genes were detected simultaneously **(B)**.

The evolved MDR and XDR-*E. coli* M19736, which were co-infected with several plasmids, were tested as donors of clinical conjugative plasmids using again as receptor *E. coli* J53 (**Figures 4A, B**). The selection was performed with ceftazidime. The evolved MDR-*E. coli* M19736 and XDR-*E. coli* M19736 strains were able to transfer the *bla*_NDM-1_ gene located in pDCASG-NDM-1 to *E. coli* J53 in the three independent biological replicates. On the other hand, when the evolved MDR-*E. coli* M19736 was used as donor, we identified two other genotypes in the transconjugants (**Figure 4A**). The first one transferred simultaneously the *bla*_NDM-1_ (pDCASG6-NDM-1) and *bla*_CTX-M-15_ (pDCAG1-CTX-M-15) genes in the transconjugants of *E. coli* J53. The second one harbored only the *bla*_CTX-M-15_ (pDCAG1-CTX-M-15) gene. Furthermore, when the evolved XDR-*E. coli* M19736 was used as donor, we were able to find another genotype in which *bla*_NDM-1_ (pDCASG6-NDM-1) and *mcr-1* (pM19736-MCR-1) genes were detected simultaneously (**Figure 4B**).

### 3.4 Isolation and characterization of EV from the native and evolved XDR-*Escherichia coli* M19736 strains

**Figure 5:**
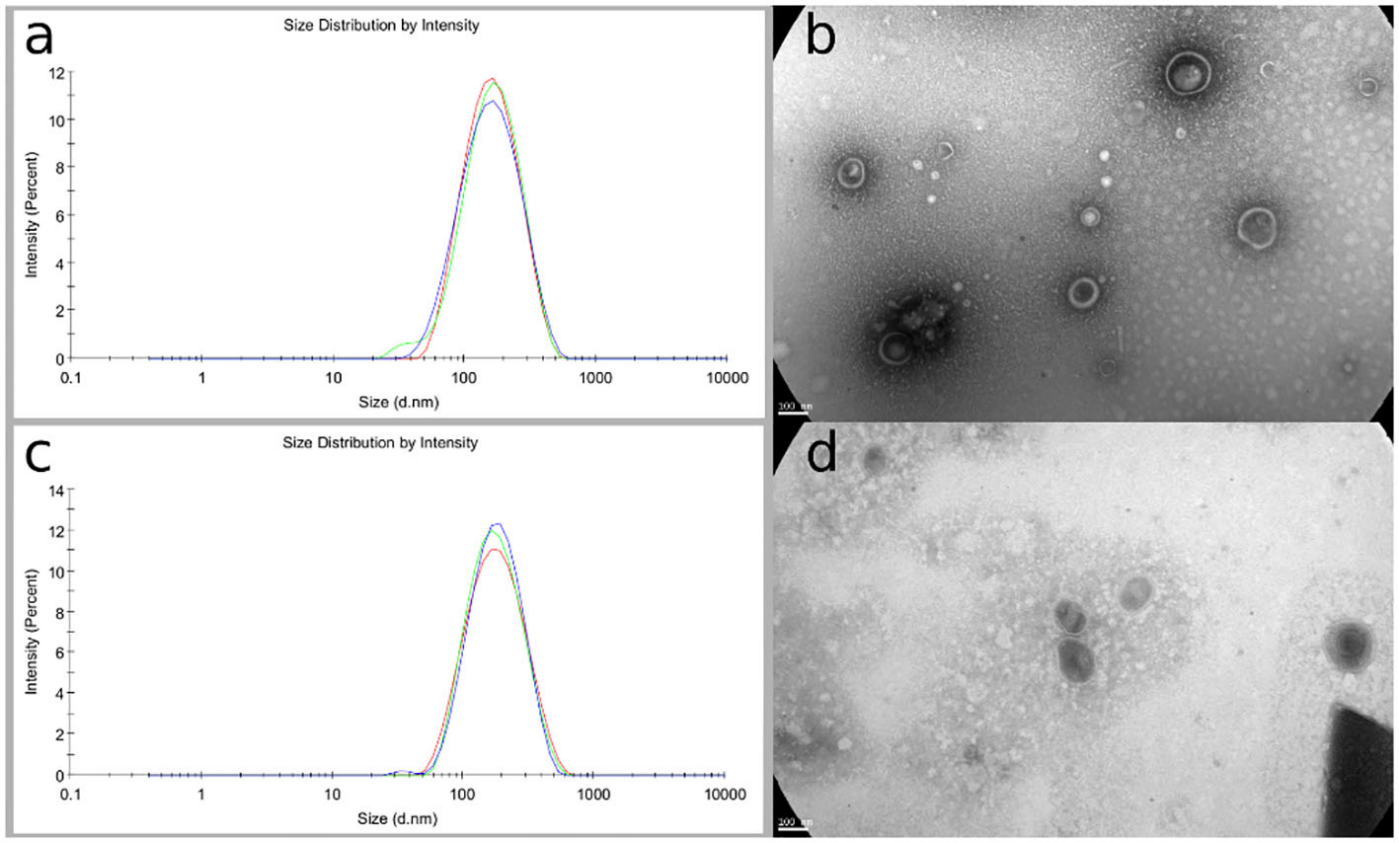
Phenotypic characterization of EV from native and evolved XDR-*E. coli* M19736. Vesicles were purified from broth cultures by ultracentrifugation and filtered through a 0.22-μm filter. DLS results are presented as the mean of three independent measurements ± SD. DLS measurement shows an average size of 193.2 ± 1.8 nm for the native *E. coli* M19736 **(A)** and 174.7 ± 0.52 nm for the evolved XDR-*E. coli* M19736 **(C)**. TEM results show in both strains EV with a double membrane, spherical in shape and heterogeneous in size **(B, D)**.

The native *E. coli* M19736 and evolved XDR-*E. coli* M19736 strains actively released EV at the log phase of growth and were isolated and collected from the supernatant broth. The cell-free EVs extracted from both strains were purified by filtration and ultracentrifugation. EVs were characterized in terms of morphology, size, and polydispersity index (PDI). The purified EVs appeared at TEM as electron-dense particles, with a spherical morphology, a bilayer membrane, and heterogeneous nanometer size (**Figures 5B, D**). The purity of the EV was confirmed as there were no bacteria visualized by TEM, and contamination controls on culture plates did not show any growth. This showed that EVs were purified successfully without contamination with other bacterial components for subsequent cell experiments. The obtained data from DLS showed that the typical diameter was approximately 193.2 ± 1.8 nm with a PDI of 0.199 ± 0.012 for the native *E. coli* M19736 (**Figure 5A**) and 174.7 ± 0.52 nm with a PDI of 0.2 ± 0.009 for the evolved XDR-*E. coli* M19736 (**Figure 5C**).

### 3.5 Content analysis of EV from both *Escherichia coli* M19736 and evolved XDR-*Escherichia coli* M19736 strains

**Figure 6:**
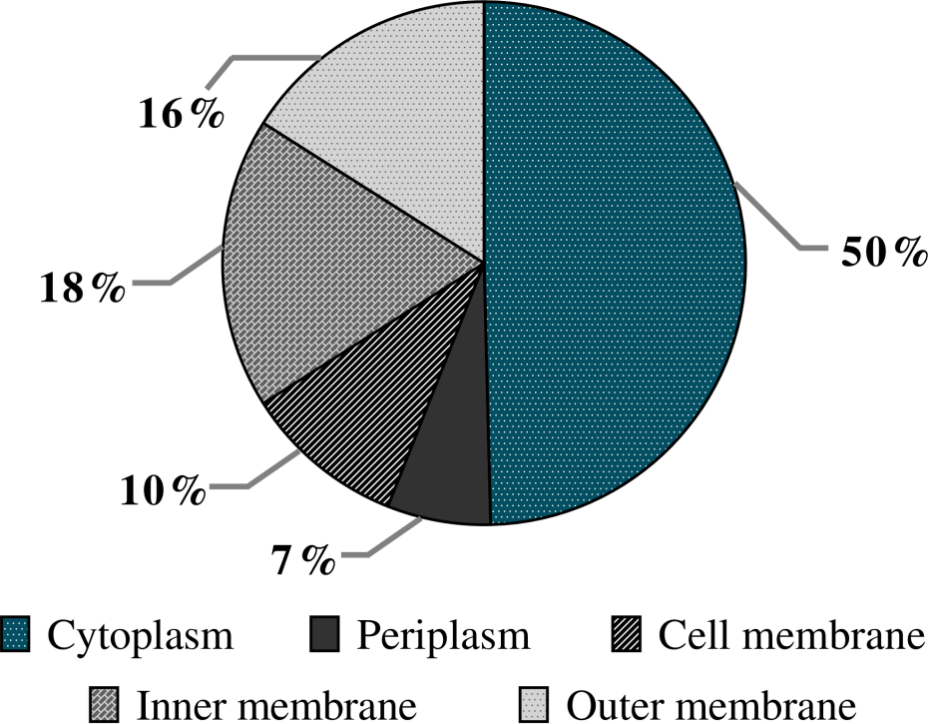
Predicted localization of EV proteins from the native *Escherichia coli* M19736. The pie diagram represents the localization of different proteins found inside or on the surface of EVs. The results are represented as the percentage of proteins found in different localizations from the native *E. coli* M19736.

**Figure 7:**
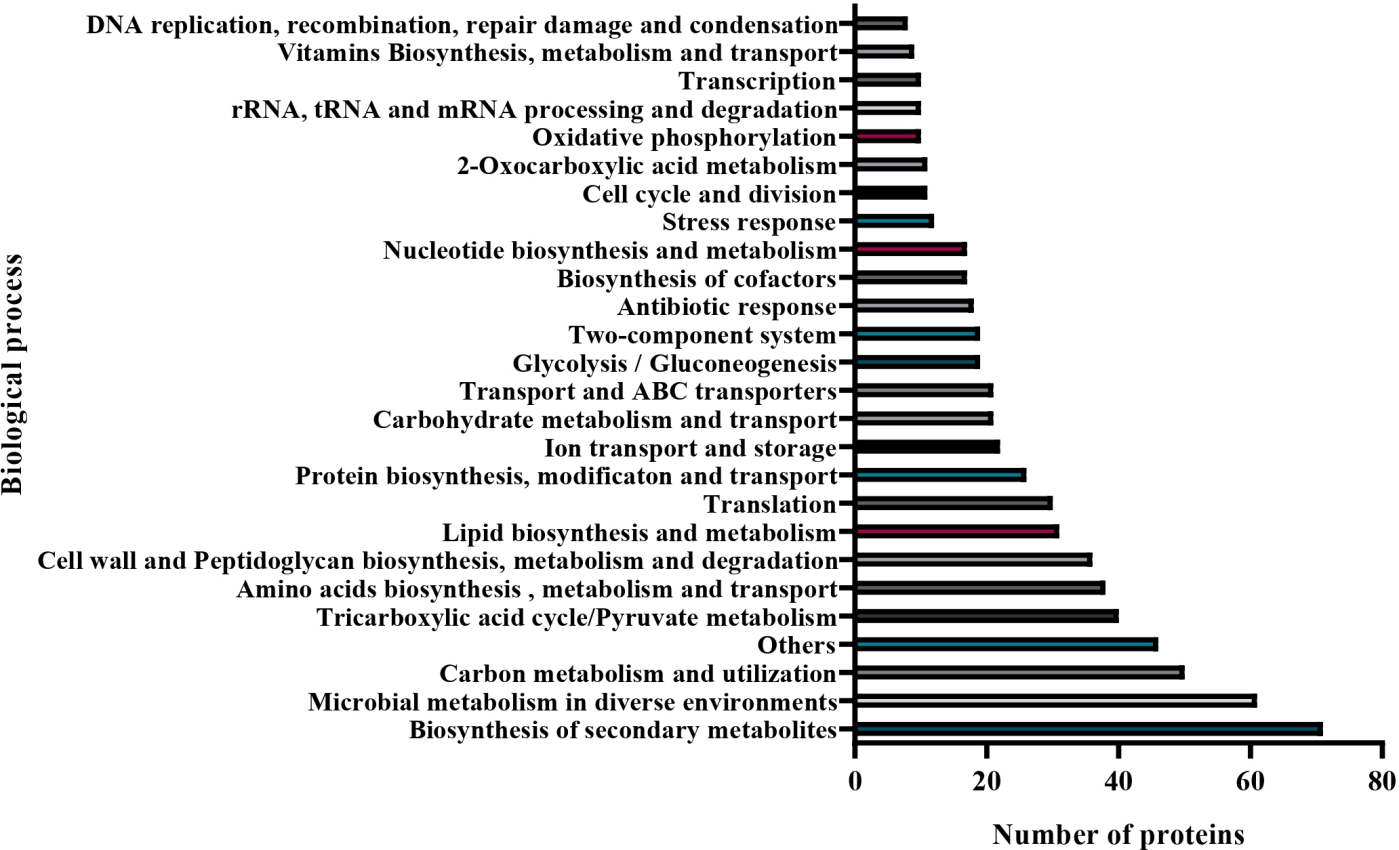
Biological processes associated with EV proteins from the native *E. coli* M19736. Bar graphs categorizing the proteins with 28 differential biological functions, with distributions in the number of proteins. The functions of EV proteins are graphed with the most abundant function at the bottom and the least abundant function at the top.

The DNA purified from EVs of the native *E. coli* M19736 gave a specific amplified product for the *mcr-1* gene. Also, EV DNA from the evolved XDR-*E. coli* M19736 allowed us to detect specific PCR products for the *bla*_CTX-M-15_, *mcr-1*, and *aadB* genes. On the other hand, the total vesicular proteins were extracted via lysis buffer and then quantified by the Micro BCA kit. The amount of protein for the native and evolved strains was 1,015 μg/ml and 1,091 μg/ml, respectively. To explore the protein contents of EV from *E. coli* M19736, LC-MS/MS analysis was applied, and 338 different proteins were identified in EVs from the native strain (**Supplementary Table 2**). Database protein was included in Vesiclepedia 2024 (http://www.microvesicles.org) (Chitti et al., 2024). Proteins were categorized into different classes including the following: cellular localization site (**Figure 6**) and biological functions (**Figure 7**). The localization of EV proteins from *E. coli* M19736 was found to be distributed as follows: 10% of the proteins were located in the cell membrane, 18% in the inner membrane, 16% in the outer membrane, 50% in the cytoplasm, and 6% in the periplasm. Moreover, among the 338 identified proteins, we were able to characterize 292 of them by biological processes/functions (**Figure 7**). Some proteins have overlapping functions. The majority were involved in the transport, metabolism, and biosynthesis of molecules such as proteins, lipids, and carbohydrates (12.9%) and biosynthesis of secondary metabolites (10.1%). Moreover, the others were involved in microbial metabolism in diverse environments (8.7%); carbon metabolism and utilization (7.1%); tricarboxylic acid cycle/pyruvate metabolism (5.7%); amino acid biosynthesis, metabolism, and transport (5.4%); cell wall and peptidoglycan biosynthesis, metabolism, and degradation (5.1%); translation (4.4%); ion transport and storage (3.1%); transport and ABC transporters (3%); glycolysis/gluconeogenesis (2.7%); two-component system (2.7%); antibiotic response (2.6%); biosynthesis of cofactors (2.4%); nucleotide biosynthesis and metabolism (2.4%); stress response (4.1%); cell cycle and division (1.7%); oxidative phosphorylation (1.4%); rRNA, tRNA, and mRNA processing and degradation (1.4%); transcription (1.4%); vitamin biosynthesis, metabolism, and transport (1.3%); and DNA replication, recombination, repair, damage, and condensation (1.1%). The main functions and pathways enriched in our EV proteins have been related to the same as other EV protein cargoes of XDR *K. pneumoniae* (Hussein et al., 2023). Other functions were also found to be represented in less than one percent such as quorum sensing, lipopolysaccharide (LPS) and lipid A biosynthesis, biofilm formation, respiratory electron transport chain, glutathione metabolism, Gram-negative bacterium-type cell outer membrane assembly, nitrogen metabolism, heme and porphyrin biosynthesis and metabolism, glyoxylate and dicarboxylate metabolism, methane metabolism, organic substance metabolism, organic acid catabolism, and chemotaxis.

Interestingly, proteins associated with the LPS biosynthesis pathway have been found, among others: the LPS assembly OM complex LptDE β-barrel component LptD and LptE (**Supplementary Table 2**), proteins that form a hetero-oligomeric complex that translocates LPS to the outer membrane and allows it to anchor to the cell wall surface (Lucena et al., 2023). Proteins that form efflux bombs, such as the three proteins that make up the tripartite efflux system AcrAB-TolC (**Supplementary Table 2**), have also been found. The AcrB and AcrA proteins, respectively, make up the inner membrane and periplasm-spanning regions, and the TolC protein component is located in the bacterial outer membrane and also pairs with subunits of other membrane pumps. In addition, we found other proteins that are involved in pathways of cationic antimicrobial peptide (CAMP) resistance such as D-transpeptidase linking Lpp to murein, N-acetylmuramoyl-L-alanine amidase AmiC, lipoprotein NlpE, and periplasmic serine endoprotease DegP (**Supplementary Table 2**). Lastly, we detected the MCR-1 protein (**Supplementary Table 2**). To the best of our knowledge, the presence of this protein in EV has not been described so far. MCR-1 mediates colistin resistance by transferring phosphoethanolamine to bacterial lipid A, thereby reducing its affinity for colistin (Li H. et al., 2021).

#### 3.5.1 PPI network of EV proteins from *Escherichia coli* M19736

**Figure 8:**
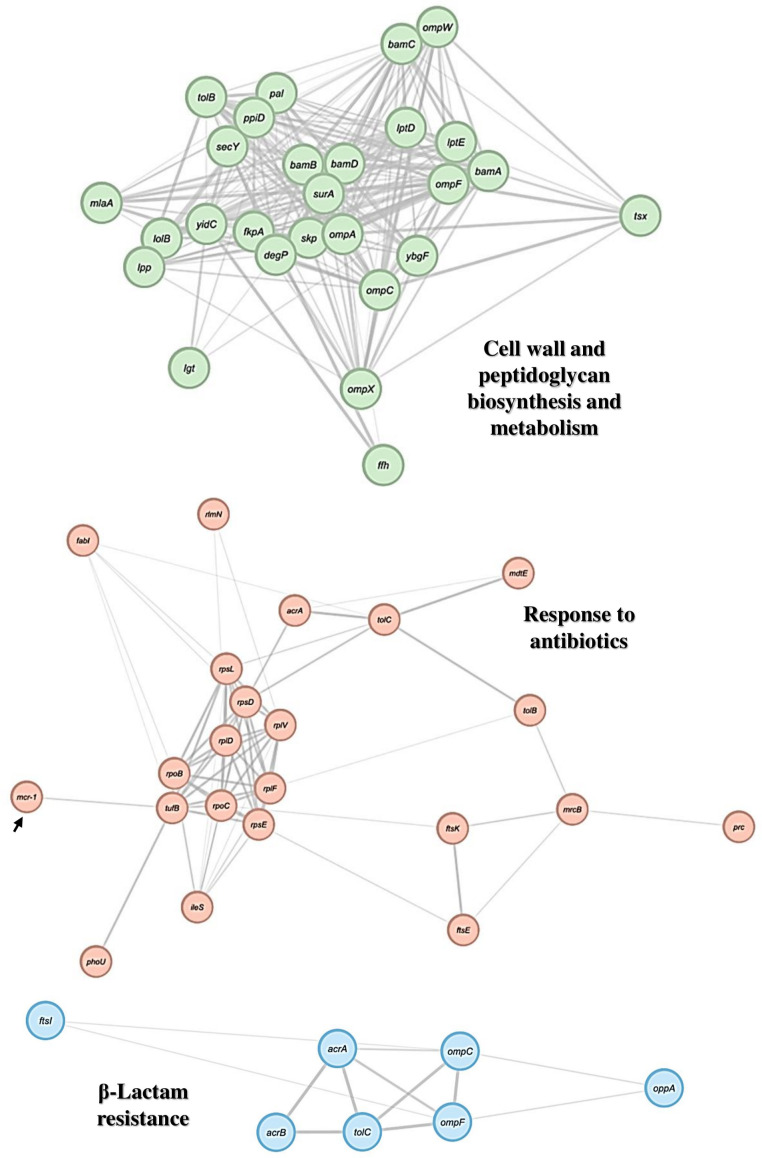
PPI network of proteins of interest found in EV from the native *E. coli* M19736. Gene Ontology and KEEG enrichment analysis of genes/proteins from the proteomes of EVs derived from the native *E. coli* M19736 using the STRING software and visualization using the Cytoscape software. The node colors represent the biological process or cellular functions of the genes/proteins of interest according to significant associations of related Gene Ontology and KEEG terms. The arrow points to the MCR-1 protein.

We constructed a PPI network using the STRING database and analyzed it using the Cytoscape software. Gene Ontology and KEEG enrichment analysis permitted to generate the network diagrams. Each node and line represented a term and the correlation between terms, respectively. The color of the terms indicates the classification of nodes based on their functions. We obtained three different PPI networks based on function/biological process/pathway found in the EV protein from *E. coli* M19736 (**Figure 8**). The first one showed cell wall and peptidoglycan biosynthesis and metabolism, represented by 27 nodes and 228 edges, with an average local clustering coefficient of 0.666 and PPI enrichment *p*-value of 1.15e−03. The second one showed an antibiotic response, represented by 23 nodes and 68 edges, and the average local clustering coefficient was 0.819 and the PPI enrichment *p*-value was 1.0e−16. The third one showed a pathway related to β-lactam resistance, represented by 7 nodes and 12 edges, with an average local clustering coefficient of 0.762 and PPI enrichment *p*-value of 1.94e−07.

Furthermore, the finding of the interaction of some specific proteins described above is of particular interest to our work. Some of these proteins could interact to generate antibiotic response/resistance, such as LptD and LptE proteins, which have been suggested to be involved in an antibiotic stress response leading to increased production and accumulation in the outer membrane (**Figure 8**) (Lucena et al., 2023). The efflux pump AcrAB-TolC is involved in conferring CAMP resistance in different bacteria, although this is controversial in *E. coli* (Blair et al., 2022). Overexpression of these efflux pumps also confers resistance to a variety of antibiotics (Brindangnanam et al., 2022). We also found several proteins involved in ribosomal and RNA degradation, including the chaperone Hsp70 (DnaK), and 30S ribosomal proteins that have been shown to interact with the MCR-1 protein (**Supplementary Table 2**) (Li H. et al., 2021). It should be noted that in addition to these proteins, the above mentioned proteins associated with CAMP resistance and the AcrA-TolC system are also important in the MCR-1 protein interactome (Li H. et al., 2021).

## 4 Discussion

HGT is a powerful force that shapes the evolution, diversification, and adaptation of bacterial communities and provides, for instance, a platform for the spread and persistence of ARGs (Piscon et al., 2023). Today, three canonical mechanisms of HGT are recognized, including transformation, transduction, and conjugation (Dubnau and Blokesch, 2019). Also, there are other non-canonical mechanisms including vesiduction, which involves secretion and uptake of EV (Li P. et al., 2022; Marinacci et al., 2023). In stressing habitats such as the nosocomial habitat, genome evolution is driven by antibiotic selection. However, there are several gaps of knowledge in this field. An area not well understood yet, it is what occurs with multidrug-resistant bacterial communities and plasmids that carry ARG in periods of time in which there is no antibiotic selection pressure. Also, the role of sporadic clones related to the spread of ARG is little studied to date. Here, we exposed the ability of a sporadic clone of *E. coli*, the *E. coli* M19736 strain belonging to ST615, harboring a plasmid with the *mcr-1* gene, to acquire a wide variety of clinical conjugative plasmids, including sequential co-infection of three conjugative plasmids harboring ARGs of current clinical interest (*mcr-1*, *bla*_NDM-1_, and *bla*_CTX-M-15_) from different species of bacteria (**Figure 1**). In turn, its competency to disseminate the conjugative plasmids again to another bacterial host was shown (**Figure 3**). At the same time, other mechanisms that were tested in this strain such as transformation for the paadB plasmid and vesiduction of native and evolved *E. coli* M19736 strains were identified that could be relevant reservoirs. *bla*_CTX-M-15_ was found in EV even if the evolved XDR-*E. coli* M19736 had rapidly lost the plasmid pDCAG1-CTX-M-15 after its acquisition, showing the essential role of EV for the dissemination of ARG in bacterial communities. Interestingly, the MICs for carbapenem antibiotics (ERT and MEM) were slightly higher in *E. coli* M19736::pDCVA3-NDM-5, evolved MDR-*E. coli* M19736, and evolved XDR-*E. coli* M19736 than the respective donor strains and transconjugants of *E. coli* J53, showing the ability of one sporadic clone to express the crucial ARG after HGT acquisition.

It has been a while since the population structure of *E. coli* has been identified; long-term stability and wide geographic distribution of individual lineages have been identified (Bobay et al., 2015). Some of these clones are pandemic, such as *E. coli* ST131, which is the predominant extraintestinal pathogenic *E. coli* that causes multidrug hospital infections, usually harboring *bla*_CTX-M-15_ and/or carbapenemases (Mathers et al., 2015; Sanz et al., 2022). In a recent retrospective epidemiological study performed with 71 relevant carbapenem-resistant *E. coli* strains isolated from 2008 to 2017 from Argentina, several pandemic clones including ST10, ST38, ST131, ST155, ST648, and ST1193 were found prevalent (Sanz et al., 2022). From this bacterial population under scrutiny, three carbapenem-resistant *E. coli* strains harboring the *mcr-1* gene were also found belonging to the pandemic clone ST10 and to sporadic clones ST12657 and ST12667. Interestingly, two of these strains (*E. coli* ECO 37 isolated in 2014 and *E. coli* ECO 81 isolated in 2015) were isolated before the first description of the *mcr-1* gene in isolates from animals, food, and patients in China (Liu et al., 2016). *Escherichia coli* ST615 was not identified in those epidemiological studies and in other studies performed with *E. coli* strains from Argentina isolated from the clinic or other environments before or after the COVID-19 pandemic (Sennati et al., 2012; Dominguez et al., 2019; Faccone et al., 2023; Gramundi et al., 2023; Piekar et al., 2023), confirming the sporadic condition of this clone. *Escherichia coli* M19736 ST615 strain isolated in 2015 was identified at that time as one of the first isolates harboring the *mcr-1 *gene in human infections caused by *E. coli* in Latin America (Rapoport et al., 2016). The importance and scope of a wide variety of sporadic clones has not yet been studied in-depth. It is likely that *E. coli* ST615 lineage could capture crucial ARG such as the *mcr-1* gene, with the ability to transfer consequently to other strains as shown in the present study and previously (Rapoport et al., 2016). We also identified that the *E. coli* M19736 ST615 strain was able to acquire a diversity of plasmids of different incompatibility groups harboring multiple ARGs from three different species (*E. coli*, *K. pneumoniae*, and *S. marcescens*) (**Figure 1A**). In addition, co-infection of plasmids sharing the same incompatibility group such as pDCVA3-NDM-5 and pIncFII-M19736 in *E. coli* M19736::pDCVA3-NDM-5 or pDCAG1-CTX-M-15 and pIncFII-M19736 in evolved MDR-*E. coli* M19736 strain was found (**Table 1**). Several differences were identified among the three IncFII replicons (**Supplementary Table 3**) that could be related, in part, to their ability to co-infect and to be maintained together during 10 days of daily subcultures by *E. coli* M19736.

Our studies showed that transconjugants of *E. coli* M19736 ST615 maintained pDCAG1-CTX-M-15 (IncFII) and pDCASG-NDM-1 (IncC) plasmids for 10 days at 100% while maintaining its native one, pMCR-M19736 (IncI2) with the *mcr-1* gene, without antibiotic pressure (**Figure 2A**). The co-infection with pDCAG1-CTX-M-15 (IncFII) and pDCASG-NDM-1 (IncC) is interesting since both have shown to possess a pandemic behavior. On one hand, the pDCAG1-CTX-M-15 plasmid has an F2:B10 replicon; IncF-type replicons have shaped the evolution of the main fluoroquinolone-resistant ST131-*H*30 clades adding an advantage resistance to several families of antibiotics including the presence of the *bla*_CTX-M-15_ gene (Johnson et al., 2016). On the other hand, pDCASG-NDM-1 belongs to the IncC group plasmids that are widely distributed among Gnb, with a large range of hosts in which these plasmids can replicate (Ambrose et al., 2018). IncC plasmids have islands of resistance incorporated in different plasmid locations where different ARGs can be accumulated including *bla*_NDM_ and *bla*_KPC_ genes (Ambrose et al., 2018; Ambrose, 2020). At first glance, since both pDCAG1-CTX-M-15 and pDCASG-NDM-1 plasmids had toxin–antitoxin systems (*CcdA/B* and *pemK/L* and *HigA/B*, respectively) and different incompatibility groups (IncFII and IncC, respectively), a low percentage of loss was expected during co-infected subcultures. Recent advances in the field profoundly questioned the role of toxin–antitoxin systems in bacterial physiology, stress response, and antimicrobial persistence (Jurėnas et al., 2022). More investigations are needed to evaluate their role in clinical isolates harboring several plasmids.

Recently, it has been shown that plasmids carrying a carbapenemase such as KPC or NDM could be efficiently conjugated to strains carrying the *mcr-1* gene and vice versa and that these plasmids could stably co-exist in clinical *Enterobacteriaceae* strains (Liu et al., 2021). Although the clonality of these strains was not determined, these results are congruent with our experiments of the evolved *E. coli* M19736 strains (**Figure 2**), which were able to transfer in turn the conjugative plasmids acquired previously. In addition, our experiments showed that when the transconjugant *E. coli* M19736::pDCAG1-CTX-M-15 was co-infected with additional plasmids, our evolved MDR and XDR-*E. coli* M19736 ST615 strains showed different patterns of ARG maintenance with the ability to keep almost at 100% the *bla*_NDM-1_ gene located in the pandemic IncC plasmid pDCASG-NDM-1.

Concerning co-infection of plasmids, it is generally overlooked that bacterial strains frequently harbor multiple plasmids, and understanding them is of utmost importance, especially for those relevant in the clinical context (Dionisio et al., 2019). Other studies have shown that bacteria can carry more than one type of plasmid; for example, it has been shown that 27 strains of *E. coli* producing extended-spectrum β-lactamase harbored multiple different plasmids (García et al., 2007). Positive epistasis between co-infecting plasmids has been shown which minimizes the cost of plasmid carriage and increases the ability of plasmids to persist in the absence of selection for plasmid-encoded traits, suggesting that epistasis may have an important role in resolving the “plasmid paradox” (San Millan et al., 2013), which is in agreement with our results. We also found that maintenance of plasmids without antibiotic selection varied depending on the plasmids that co-infected the host (**Figure 2**), such as the case of the evolved MDR-*E. coli* M19736 and XDR-*E. coli* M19736 strains related to the maintenance of pDCAG1-CTX-M-15 (**Figure 3**). The addition of plasmid paadB (Inc15A) triggered the loss of pDCAG1-CTX-M-15 from the evolved XDR-*E. coli* M19736 strain. Concerning this, a strong co-evolution has been shown between some *E. coli* ST131 lineages and specific plasmids including F2:B10 (Ny et al., 2019), which could explain in part its ease of getting lost in a sporadic clone such as *E. coli* ST615.

In order to follow the trajectory of plasmids that were co-infecting our evolved strains, conjugation assays were performed revealing that the evolved MDR and XDR-*E. coli*-M19736 strains were able to transfer one or two plasmids simultaneously while keeping always the pM19736 plasmid (**Figure 4**). Previous plasmid co-transfer in *E. coli* strains showed that when hosts harbor two, three, or four distinct plasmids, the co-transfer of both plasmids tends to be limited by the plasmid exhibiting the lowest conjugation rate (Gama et al., 2017; Darphorn et al., 2022). In disagreement with this, we did not find significant differences between the conjugation efficiencies of these plasmids (**Figure 1B**), so in the cases where the co-transfer happens, we cannot associate with the conjugation rate of each plasmid (**Figure 4**). Also, our results are congruent with the hypothesis that suggests that de-repression could occur simultaneously on co-resident plasmids. In fact, this could happen as a response to a common stimulus. The idea that a common stimulus triggers the transfer of one or the other plasmid could explain why one or the other plasmid is co-transferred as proposed by Gama et al. (2017). However, it is not clear in our study what stimulus could have triggered co-transfection in our experiments, but selection with ceftazidime may be studied deeply. The tendency of bacteria to maintain several plasmids simultaneously independently of the antibiotic pressure imposed on the pathogens has been shown (San Millan et al., 2013). There are two predictable ways for a strain to harbor multiple plasmids: i) through sequential plasmid acquisition as we showed in the experiments using the sporadic clone *E. coli* M19736 as the recipient strain that evolved to MDR-*E. coli* M19736 and XDR-*E. coli* M19736 strains (**Figure 1A**) and ii) by simultaneous transmission of several plasmids as we showed in the experiments that we performed using the evolved MDR and XDR-*E. coli* M19736 strains as donors to *E. coli* J53 (**Figure 4**). Our results showed that one strain belonging to a sporadic clone is able to be co-infected with several plasmids in both ways as previously suggested (Dionisio et al., 2019).

Since recent research has revealed that EVs may play an important role as a reservoir of genes and proteins associated with AMR contributing to this global threat through several other mechanisms (Ciofu et al., 2000; Rumbo et al., 2011; Lee et al., 2013; Chatterjee et al., 2017; Bielaszewska et al., 2020; Marchant et al., 2021), we also investigated the ability of the EV of the *E. coli* M19736 strain to harbor AMR determinants. First, we analyzed the EV of *E. coli* M19736 and evolved XDR-*E. coli* M19736 strains by PCR searching for the ARG. We found in both strains the presence of the *mcr-1* gene which has been also previously described (Li X. et al., 2021). Interestingly, EVs from the evolved XDR-*E. coli* M19736 strains harbored *bla*_CTX-M-15_ though the pDCAG1-CTX-M-15 was previously lost as shown by WGS analysis and maintenance experiments, suggesting that EVs could be a relevant reservoir of ARG for susceptible bacteria. Unexpectedly, we did not find the *bla*_NDM-1_ gene in the EV content of the evolved strain by DNA PCR. This result was surprising because the *bla*_NDM_ gene is the most described gene reported in EV so far (Chatterjee et al., 2017; Li X. et al., 2021). These findings lead us to consider what might play a role in DNA cargo selection in EV, as the mechanism by which DNA cargo is enriched or excluded from EV remains unclear (Zavan et al., 2023). Plasmid type has been shown to influence gene loading (Tran and Boedicker, 2017). However, this is not the only reason explaining why *bla*_NDM-1_ located in pDCASG6-NDM-1 was not found in EV. Other features could be involved in the selection of DNA loading in EV, such as the location of each type of plasmid within the cell (Pogliano, 2002). Currently, there are more studies focusing on the effects of growth stage on EV selection load. For example, analysis of EV produced by *Helicobacter pylori* reveals that bacterial growth stage affects the size, composition, and selection of protein load (Zavan et al., 2019). Other research showed that plasmids belonging to different incompatibility groups bind to different sites within the cell (Pogliano, 2002). Aktar et al. (2021) also performed some experiments to evaluate the incorporation of plasmid DNA into EV. The results showed that peptidoglycan defects increased plasmid sorting into EV. It is well known that the use of cell wall antibiotics increases EV production (Bauwens et al., 2017; Lucena et al., 2023). In summary, since EV DNA packaging is not a random process, more work is needed to understand the rules of plasmid packaging into vesicles.

Furthermore, in EVs from the *E. coli* M19736 strain, the proteins from the LPS biosynthesis pathway were found (**Supplementary Table 2**), and 2-dehydro-3-deoxyphosphooctonate aldolase (KdsA) is responsible for linking lipid A and core oligosaccharides through the synthesis of other proteins (Hussein et al., 2023). The experiments of Strohmaier et al. (1995) demonstrate that KdsA undergoes growth phase-dependent regulation at the transcriptional level, which again supports the idea that the growth phase is related to the selection of cargo in EV. Also, this protein increases following polymyxin B treatment (Hussein et al., 2023) which supports the idea that treatment with each antibiotic could collaborate in the selection of load in EV. Also, in EV from the *E. coli* M19736 strain, the MCR-1 protein was found (**Supplementary Table 2**). To our knowledge, the presence of this protein has not been previously described. It has been demonstrated that MCR-1-interacting proteins were mainly involved in ribosome and RNA degradation such as the 30S ribosomal subunit proteins S5 and S10 (Li H. et al., 2021). These proteins were also identified in our EV extractions and among other ribosomal proteins as well (**Supplementary Table 2**). Also, the two-component AcrA-TolC multidrug efflux pump interacts with the MCR-1 protein and is involved in *mcr-1*-mediated colistin resistance (Li H. et al., 2021). In addition, EVs have shown to have an effect of protection of colistin’s administration over other strains (Marchant et al., 2021) that could apply to the *E. coli* M19736 strain under scrutiny in the present work. For relevance in the nosocomial niche, the main functions and pathways enriched in our EV proteins were related to EV protein’s cargo of XDR *K. pneumoniae* strains as previously shown (Hussein et al., 2023).

Since a few clinical sporadic clones have shown to be prone to accept plasmids by transformation (Ramirez et al., 2011), deeply analyzing the genomic plasticity of *E. coli* M19736, artificial competency was also achieved. This assay evidenced the ability of this strain to accept the paadB plasmid by chemical competency of the native and evolved MDR-*E. coli* M19736 strains, which could be maintained by *E. coli* M19736 during at least 58 generations (**Figure 2**). Furthermore, sporadic clones were found to capture relevant features of environmental plasmids that later could spread within the nosocomial niche such as the case of pDCPR1 which has a mosaic structure with parts of successful phytopathogen plasmids such as pIII from pathogenic *Xanthomonas albilineans* and pPSV48C from another pathogenic species *Pseudomonas savastanoi* (Vilacoba et al., 2014). This ability to capture genetic platforms from the bacterial communities of the open environment adds a relevant role to sporadic clones in the framework necessary for the success of AMR in the nosocomial niche.

In summary, our studies showed that *E. coli* M19736 strains belonging to the sporadic clone ST615 have a range of valuable tools for survival under antibiotic pressure as we identified its genomic plasticity to acquire plasmids from various species with crucial ARGs, with proficiency to keep them and disseminate in turn by conjugation to other strains. Furthermore, its competence to produce EVs containing several valuable AMR determinants with the potential to be disseminated and its ability to receive plasmids by transformation as demonstrated in the present study indicated a great adaptation to the in-hospital environment. Based on this, sporadic clones could play a crucial role in a clinical context by adapting to different selection pressures and at the same time by spreading plasmids to other bacteria which guarantees an important link in the chain of events that leads bacteria to resist in the nosocomial niche.

## Data availability statement

The *E. coli* M19736 genome data presented in the study is deposited in GenBank under the accession numbers PRJNA1080650 and PRJNA1102182. The assembled plasmids pDCASGNDM-1 and pDCAG1-CTX-M-15 were deposited in GeneBank under the accession numbers PP622803 and PP632099. The data base protein of EV was included in Vesiclepedia 2024 (http://www.microvesicles.org) study ID 3592.

## Author contributions

LCP: Data curation, Formal analysis, Investigation, Methodology, Visualization, Writing – original draft, Writing – review & editing. MO: Conceptualization, Investigation, Project administration, Writing – review & editing. AG: Data curation, Writing – review & editing. TP: Investigation, Methodology, Writing – review & editing. AA: Data curation, Investigation, Writing – review & editing. MQ: Conceptualization, Data curation, Formal analysis, Funding acquisition, Investigation, Resources, Supervision, Writing – review & editing. DC: Conceptualization, Formal analysis, Funding acquisition, Investigation, Project administration, Resources, Supervision, Writing – original draft, Writing – review & editing, Validation.
